# School Climate Questionnaire: A New Tool for Assessing the School Environment

**DOI:** 10.3389/fpsyg.2022.871466

**Published:** 2022-07-01

**Authors:** Alexandra A. Bochaver, Aleksei A. Korneev, Kirill D. Khlomov

**Affiliations:** ^1^Centre for Modern Childhood Studies, Institute of Education, National Research University Higher School of Economics, Moscow, Russia; ^2^Faculty of Psychology, Lomonosov Moscow State University, Moscow, Russia; ^3^Institute for Social Sciences, Russian Presidential Academy of National Economy and Public Administration, Moscow, Russia

**Keywords:** school climate, school climate measures, school environment, school students' wellbeing, school norms

## Abstract

The school environment is of great importance for the socialization of children. At school, children learn many values, rules, and skills that relate to building relationships that include friendship, support, and competition. The school largely shapes children's behavior and expectations from society in the future. This study validates the new 22-item School Climate Questionnaire (SCQ) using a sample of Russian school students. A total of 4,776 respondents from 9 to 18 years old participated in the correlational study and filled the online-survey that includes SCQ. The Revised Olweus Bully/Victim Questionnaire, the Warwick–Edinburgh Mental Well-Being Scale (WEMWBS), and the Academic Motivation Scale were used to examine the convergent validity of the SCQ. Two hypotheses were examined that the new tool SCQ has structural validity (three factors) and convergent validity (which is proven by the significant correlations with victimization, aggression, mental wellbeing, and academic motivation). According to confirmatory factor analysis (CFA), it was shown that the questionnaire has three factors; their reliability is satisfactory. As expected, the results revealed significant correlations between the three scales of SCQ and scales of Victimization, Aggression, Mental Well-Being, and different types of Academic Motivation. The SCQ is a reliable and valid instrument that may be recommended for use by researchers and practitioners in different areas of educational psychology.

## Introduction

The school environment is of great importance for the socialization of children. In addition to academic learning, children learn many values, rules, and skills that relate to building relationships, academic motivation, behavioral patterns, etc. The school largely shapes children's behavior and expectations from society in the future (Crosnoe, 2011). Many studies have been dedicated to the different aspects of a school as a complex social system, which proposes to its “inhabitants” certain values, beliefs, and attitudes (Olsen et al., 2018). From the perspective of bioecological systems, school is an important component of the microsystem (Bronfenbrenner, 1979; Bronfenbrenner and Morris, 2006). The interactions between the child and school environment (proximal processes) are durational and influential; they are bi-directional, which means that the child influences school, as well as school influencing the child. The proximal processes affect development most directly, but they themselves are influenced by the nature of the person, the context, and the time (Ashiabi and O'Neal, 2015).

The main construct used to describe these characteristics is usually the *school climate*. The school climate is an intensively developed, complex, broad, and multifaceted construct that draws upon a variety of cultural, contextual, perceptual, and behavioral factors (Bradshaw et al., 2021). There is no universally accepted definition for school climate, but predominantly it is understood as the “*quality and character of school life. School climate is based on patterns of people's experiences of school life and reflects norms, goals, values, interpersonal relationships, teaching and learning practices, and organizational structures*” (Cohen et al., 2009, p. 182).

As Grazia and Molinari (2021) suggest, the construct of the school climate should be supplemented by three features: (1) comprehensiveness and multidimensionality of the construct, such as academic experience, relations, safety, and institutional environments; (2) its impact on the various outcomes (e.g., academic achievement, psychological wellbeing, level of bullying, and behavioral misconduct); and (3) the flexibility and potential of the tool, which may be changed *via* interventions. “*By and large, school climate emerges as a useful access route to promote students' and teachers' self-reflection that eventually foster school change and improvement*” (Grazia and Molinari, 2021, p. 562).

There are different models of the school climate, which include a different number of levels, from exclusively school students' perceptions up to a cultural-ecological model of school climate. Bronfenbrenner's ideas suggest that perceptions of school climate are associated with complex personal, cultural, and contextual factors (La Salle et al., 2015). Although the focus of the school climate is usually on the interpersonal and relational aspects, it is often expanded beyond them and incorporates issues related to safety, support, and engagement (Bradshaw et al., 2021). There is an agreement that the “subjective” nature of the school climate dominates over the “objective” school facilities (Thapa et al., 2013), and these perceptual aspects of the school climate play a more central, critical role in influencing behavior than the objective elements of the school (O'Brennan and Bradshaw, 2017; Bradshaw et al., 2021). The broad dimensions of the school environment, which shape the school climate and are usually discussed from the perspective of their expanded impact, are *safety, relationships, teaching and learning*, and *environment-structure* (Cohen et al., 2009; Thapa et al., 2013; La Salle et al., 2015). *Safety* includes both physical and social-emotional safety experienced by students, a supportive environment, and clear rules. *Relationships* include norms related to respect for diversity and collaboration, supportive relationships with teachers, and school connectedness. *Teaching and Learning* encompasses the quality of instruction—students' and staff members' participation in and shaping of educational goals, expectations for student achievement, praise and reinforcement, and social, emotional, and ethical learning and instruction. *Environment-structure* refers to the physical environment at school, i.e., the maintenance and functioning of facilities and the aesthetic aspects (Cohen et al., 2009; Thapa et al., 2013; Wang and Degol, 2016; Capp et al., 2020). The US Department of Education's model of school climate focuses on three very similar facets of school climate that include *safety, engagement*, and the *environment* (U.S. Department of Education Office of Safe Healthy Students, 2016; Bradshaw et al., 2021).

Many studies show that school climate is functionally and directly associated with school safety, other positive indicators of success and wellbeing of a student (Aldridge et al., 2016; Lombardi et al., 2019) and inversely related to problem-focused outcomes, violence, risk, and problem behaviors (Capp et al., 2020; Bradshaw et al., 2021). This is confirmed both by correlational and longitudinal studies. This is one of the most important reasons for studying its characteristics and developing solutions to improve it.

A negative school climate is characterized by students' feelings of alienation from teachers, lack of peer and teacher support, and perceived tolerance of bullying (Ebbert and Luthar, 2021). Negative aspects of the school climate are associated with a number of negative behavioral indicators, such as absenteeism, truancy, dropout, suspension, drug use, and aggressive behavior (Thapa et al., 2013; Wang and Degol, 2016; Berkowitz et al., 2017; Bradshaw et al., 2021). Evidence shows that the school climate influences bullying and victimization in multiple ways (Gottfredson et al., 2005; Hong and Espelage, 2012; Astor et al., 2013). In many studies, negative experiences with school climate are associated with increased bullying and victimization (Kosciw et al., 2011; Hong and Espelage, 2012) and internalizing and externalizing symptoms (Ebbert and Luthar, 2021). Reaves et al. (2018) in a meta-analysis of longitudinal studies show a small but significant relationship between school climate and problem behavior (violence, bullying, and later school delinquency) over time. School norms play important roles in the wellbeing of students. For example, the results of the study of the involvement of school children in the extracurricular activities indicate that for boys breaking school social norms is associated with experiencing victimization, whereas for girls breaking gender norms increases their likelihood of being victimized (Berger et al., 2022).

A positive school climate includes having a caring adult at school and supportive relationships with teachers, norms of respect for diversity, and perceived peer support (Volk, 2020; Ebbert and Luthar, 2021). Thus, a positive school climate is positively associated with academic achievement (Astor et al., 2013; Berkowitz et al., 2017; Demirtas-Zorbaz et al., 2021; Ebbert and Luthar, 2021), academic self-efficacy (Zysberg and Schwabsky, 2021), and academic motivation, which is the driving force behind student academic performance (Volk, 2020; Wang et al., 2020). A positive school climate has also been associated with reduced violence and victimization in schools (Astor et al., 2002; Espelage and Swearer, 2003; Farina, 2019) and decreased student delinquency and substance abuse (Zullig et al., 2010; Thapa et al., 2013). Decreased dropout rates (Freudenberg and Ruglis, 2007) and improved math achievement (Berkowitz et al., 2015) are also linked to a positive school climate (Capp et al., 2020). A positive school climate can help to mitigate the frequency and impact of bullying and victimization (Birkett et al., 2009).

There are many English-language tools for assessing the school climate. Particularly, as the most available and appropriate for school staff to use, and having the best indicators of reliability (Olsen et al., 2018) are the Comprehensive School Climate Inventory (National School Climate Center, 2021), School Climate Assessment Instrument (Alliance for the Study of School Climate, 2021), California School Climate, Health, and Learning Survey (WestEd, 2022), and Meriden School Climate Survey (Gage et al., 2016). They have different factor structures and include a different number of scales. All these measures assess *interpersonal relationships, safety/perception of the environment of the school*, and the different additional indicators, such as teaching and learning, institutional environment, and social media (Olsen et al., 2018).

For the Russian context, the issue of assessing the school climate is very relevant. Russian school education has been in a process of permanent reform for many decades. The class in Russian schools is a stable group, which includes about 25–30 students grouped according to the age who study mainly according to one program and one schedule (excluding a few electives, learning foreign languages in subgroups, and, in rare cases, studying according to an individual plan in some schools of large cities). In small settlements, where children rarely move in schools, the composition of the class generally does not change from the first to the eleventh grade, which makes the issue of the quality of the school climate very important. In accordance with the 2012 reforms, in the large cities, individual schools were integrated into large “educational complexes,” that include up to 20 buildings, to create uniform educational conditions. However, they continue to have strong differences in their educational achievements, climate, and reputation, i.e., within different buildings of the same school (Khlomov et al., 2021). There is still no reliable instrument in Russians to assess the characteristics of the school environment to correlate or predict school students' wellbeing and school engagement or in contrast, school violence, bullying, and other forms of disruptive behaviors.

The goal of this paper was to describe the development of a tool that assesses aspects of the school climate and is validated on a sample of Russian school students. We based our study on the theoretical assumptions that suggest school climate is a complex multidimensional construct, which describes the subjective perceptions of a holistic school environment and has strong associations with students' subjective wellbeing and behavior (Cohen et al., 2009; Thapa et al., 2013). We chose not to validate an international questionnaire but to develop a new tool, which would be relevant to Russian culture-specific post-Soviet educational contexts. There are different terms, such as *atmosphere, feelings, tone, ethos, occupational health, organizational health, setting, milieu, culture, and conditions for learning*, that are often used by the researchers and practitioners to characterize school climate (Bradshaw et al., 2021, p. 222). We chose the term *climate*, but we did not claim here to have assessed all the complicated components of the school climate and we are aware of the limitations of our tool; particularly, our measures focus only on the students' self-reported perceptions of the school climate, as in most studies on this topic (Cohen et al., 2009; Thapa et al., 2013; Berkowitz et al., 2017). The perspectives of teachers and other staff members are missing from the current understanding of the school climate, despite the fact that staff members are responsible for responding to bullying and violence, and for interactions and decisions that contribute to the school climate (Yoon et al., 2016; Olsen et al., 2018).

The tool corresponds to two main dimensions of the school climate, safety and relationships, and aimed to assess three indicators of them: the level of externalizing problem behavior (scale Deviant Behavior); the level of subjective unsafety and indicators of bullying (scale Subjective Unsafety); and the level of comfort experience and respective relationships (scale School Well-Being).

Hence, we hypothesized that the School Climate Questionnaire (SCQ) has the following:

(H1) structural validity (includes three scales);
(H2) convergent validity is expressed in the significant correlations between the three SCQ scales and the indicators of mental wellbeing, different types of the academic motivation, and the scales of victimization and aggression.

## Materials and Methods

The first version of this questionnaire was developed by a team of researchers and experts in 2014 to assess students' subjective safety and risk of bullying at school. It included 48 items and four scales (wellbeing, equality, unsafety, and disunity) and showed satisfactory levels of reliability and convergent validity (Bochaver et al., 2015). We formulated the items based on expert observations of the school reality and their knowledge of the implicit indicators of the school students' subjective wellbeing or unhappiness. Later, the questionnaire length, psychometric properties, and COVID-19-related issues pandemic led to the need to revise the tool. At the stage of revision, two focus groups with the adolescents (in total 21 respondents 12–17 years old, 12 women and nine men) were conducted to discuss the individual items in terms of their clarity and correspondence to the reality of the pandemic period. From the initial 48 items, 26 were removed because of their ambiguity, irrelevance (e.g., items about school trips), or their unsatisfactory psychometric properties.

### Participants and Procedure

A total of 4,776 respondents from 9 to 18 years old participated in the correlational study (M_age_ = 13.63, SD_age_ = 1.80; 2,728 women, M_age_ = 13.70, SD_age_ = 1.81 and 2,048 men, M_age_ = 13.54, SD_age_ = 1.79).

We used a convenience sampling strategy; data were collected in a series of different research projects, so the sample sizes for different tools differed. All participants were school students from the different Russian regions, i.e., Amur Region, Belgorod Region, Vladimir Region, Voronezh Region, Ivanovo Region, Irkutsk Region, Kaliningrad Region, Kemerovo Region, Kirov Region, Krasnodar Territory, Krasnoyarsk and Krasnoyarsk Territory, Lipetsk Region, Moscow and Moscow Region, Murmansk Region, Nizhny Novgorod Region, Penza Region, Perm Region, Pskov Region, Komi Republic, Republic of Sakha (Yakutia), Rostov Region, Saratov Region, Sverdlovsk Region, Smolensk Region, Tambov Region, Khabarovsk Territory, Chelyabinsk region, and The Chuvash Republic. The survey was also conducted in schools in Sevastopol and the Republic of Crimea that, regardless of political issues, have been teaching Russian-speaking children according to Russian educational curricula since 2014.

Data were collected online, conducted by classroom teachers in 2021 using 1ka.si (https://www.1ka.si). Data collection took place during an aggression prevention project implemented by the Institute of Study of Childhood, Family, and Education of the Russian Academy of Education (Moscow, Russia). Schools had participated in the project as experimental sites. The parents of the students provided their written consent to the survey of the children and to publish the results anonymously.

### Instruments

We used SCQ as the main tool, and three additional measures were included in this study to assess the convergent validity of SCQ, as they included concepts familiar to the school climate and have been already adapted for Russian culture. SCQ consists of 22 items, which are the statements of the different elements of the school environment. Participants were asked to estimate each of the items on a 2-point scale (yes/no). Example of items are as follows: “You generally like your school, it is comfortable, and it is interesting.” The original and translated items are presented in Appendix 1.

The English-language *Revised Olweus Bully/Victim Questionnaire* was developed by Breivik and Olweus (2015) and was adapted for Russia by Bushina and Muminova (2021), which includes two scales: Victimization and Aggression, each of them includes eight items on a five-point scale. Example of items: “I was called mean names, was made fun of, or teased in a hurtful way.”

The *Warwick–Edinburgh Mental Well-Being Scale (WEMWBS)* was developed by Tennant et al. (2007) and adapted in Russia by Nartova-Bochaver (Robinson et al., 2013), which is a unidimensional scale to measure self-reported mental wellbeing of respondents, it consists of 14 items on a five-point scale. Example of items: “I've been dealing with problems well.”

The *Academic Motivation Scale* was developed by Vallerand et al. (1992), adapted for Russia by Gordeeva et al. (2014), and modified for the school age by the authors. It includes 28 items on a five-point scale referred to seven scales measuring three types of intrinsic motivations (Intrinsic Motivation to Know, to Accomplish Things, and to Experience Stimulation), three types of extrinsic motivations (External, Introjected, and Identified Regulation), and Amotivation, describing the reasons for learning. Example of items: “Because I experience pleasure and satisfaction while learning new things.”

### Data Analysis

The responses of all participants from different studies were aggregated in one database and were analyzed as one dataset. The reliability was tested by Cronbach's alpha (Cronbach, 1951). To confirm the factorial structure of SCQ (H1), we used confirmatory factor analysis (CFA) (maximum-likelihood method). For the investigation of correlations between SCQ and other indicators of school students' wellbeing (H2, convergent validity), we used Pearson's correlation. The statistical analysis was conducted in R environment version 4.1.1 (R Core Team, 2020). Reliability analysis was conducted using psych package version 1.9.12.31, the CFA was conducted using lavaan package version 0.6-10 (Rosseel, 2012).

## Results

### Descriptive Statistics

Descriptive statistics of the scales are presented in Table 1.

**Table 1:** Descriptive statistics of the scales.

**Scale**	**Mean**	**Standard** **deviation**	**95% Confidence** **interval**	**Skewness**	**Kurtosis**
Deviant Behavior	3.654	2.125	(3.594; 3.714)	0.116	−0.778
School Well-being	5.593	1.766	(5.543; 5.643)	−0.728	0.099
Subjective Unsafety	1.209	1.451	(1.168; 1.251)	1.091	0.180

**Figure 1:**
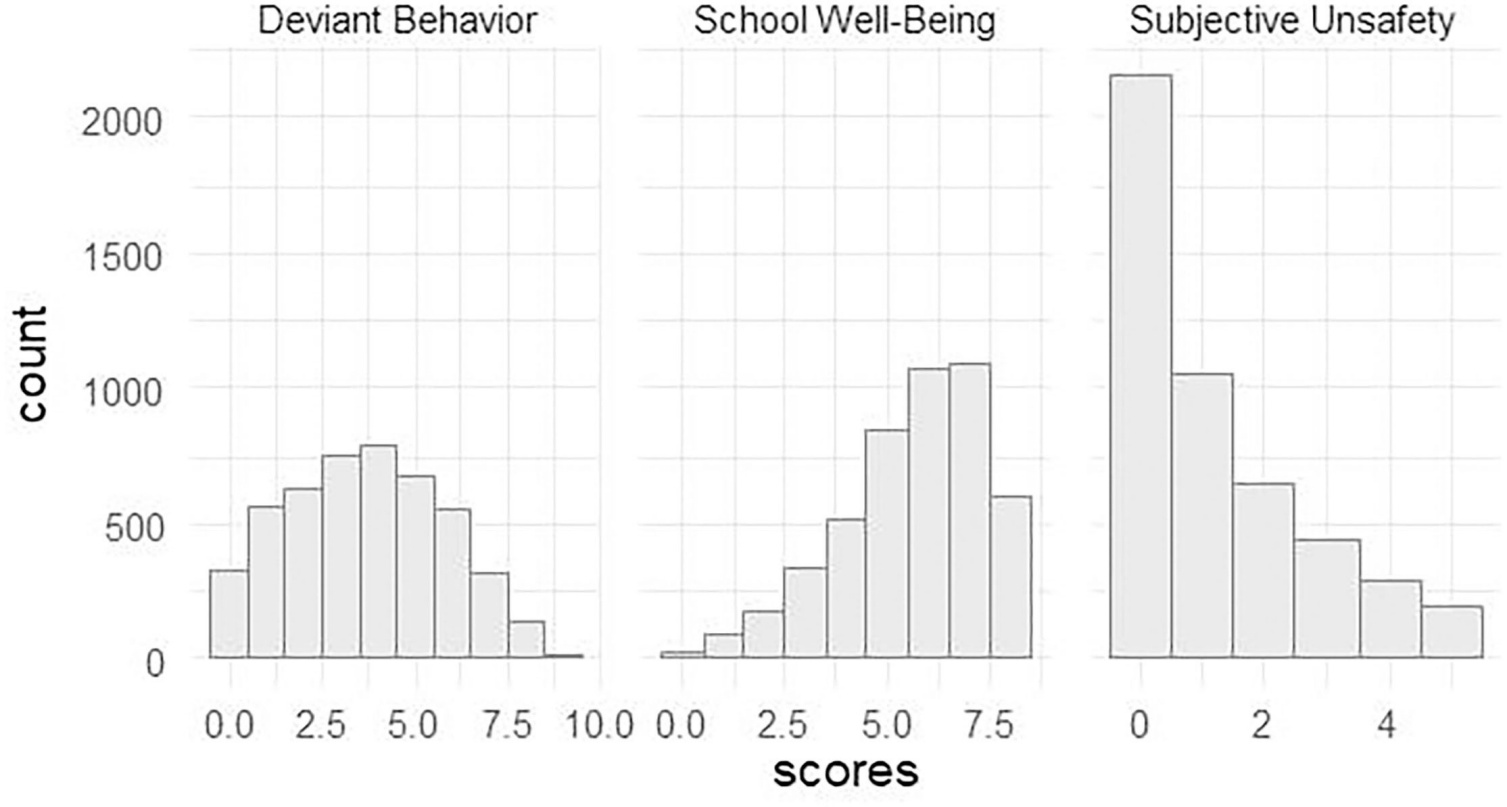
Distribution of the scores of the scales.

The distribution of the scales is depicted in Figure 1.

The distribution of the School Well-Being scale is negatively skewed, which reflects more frequent answers near the upper scale pole, hence the sensitivity of the scale is higher in its lower part. The Deviant Behavior and, especially, Subjective Unsafety scales, have positive skewness, that is, they are more sensitive on the higher part of the scales.

### Reliability

We tested reliability of the scales by Cronbach's alpha. Cronbach's alpha with 95% confidence interval (CI) was 0.62 (0.61; 0.64) for Deviant Behavior; 0.59 (0.58; 0.61) for School Well-Being; and 0.72 (0.71; 0.74) for Subjective Unsafety.

In addition, we estimated the changes of Cronbach's alpha if any item was deleted. The results show that reliability would increase if the item “*I try not to carry valuables to school at all*” is deleted from the scale Deviant Behavior (up to 0.66) and if the item “*When there is a fight at school, you don't pay attention, it's a common thing*” is deleted from the scale Subjective Unsafety (up to 0.73). Deleting any other item leads to decreasing reliability. We decided to leave the mentioned two items due to their meaningfulness according to the focus groups conducted with the adolescents.

### Factor Analysis

We used CFA to test the structure of the scale. We used the weighted least squares means and variance adjusted (*WLSMV*) estimator, which is robust to non-normally distributed variables and is better for modeling categorical or ordered data (Brown, 2006). The model included three factors (scales), which can correlate with each other. The fit indices of the model are good: χ^2^(206) = 2069.759, *CFI* = 0.931, Tucker-Lewis Index (*TLI*) = 0.922, root mean square error of approximation (*RMSEA*) = 0.048 (0.046; 0.050), and standardized root mean squared residual (*SRMR*) = 0.050. The standardized coefficients of the model are presented in Table 2.

**Table 2:** Factor loadings in the model.

**Factor (scale)**	**Item**	**Factor loadings (st.Errors)**	***p*-value**
Deviant Behavior	There's someone in your class that even a teacher can't handle	0.662 (0.015)	<0.001
	At your school, swearing sounds during recess in personal conversations	0.856 (0.011)	<0.001
	In your school, swearing is not accepted at all	−0.748 (0.013)	<0.001
	In your school, they smoke in the lavatories, under the stairs	0.616 (0.016)	<0.001
	In your school, the walls, furniture are covered with writing, stained	0.563 (0.018)	<0.001
	If someone starts yelling, fighting, the class ≪go nuts≫, what does it take to make it stop? The director should come	0.354 (0.02)	<0.001
	If someone starts yelling, fighting, the class ≪go nuts≫, what does it take to make it stop? It will stop when everyone gets tired	0.295 (0.02)	<0.001
	Your class has a reputation of bullies	0.601 (0.017)	<0.001
	I try not to carry valuables to school at all	0.058 (0.021)	0.006
School Well-Being	In your class, it is customary to have fun together after the lessons	0.402 (0.021)	<0.001
	In your class, it is customary to stand up for your own	0.663 (0.021)	<0.001
	In your class, it is customary not to interfere with each other to do what you want	0.498 (0.021)	<0.001
	There is someone in your class that everyone respects	0.353 (0.022)	<0.001
	If someone starts yelling, fighting, the class ≪go nuts≫. What does it take to make it stop? One of the students should say ≪That's enough≫	0.626 (0.019)	<0.001
	You generally like your school, it's comfortable, there is interesting	0.742 (0.016)	<0.001
	You don't like school in general, it's uncomfortable, no one is friends with anyone	−0.802 (0.02)	<0.001
	Your class has a reputation of the honor students	0.37 (0.021)	<0.001
Subjective Unsafety	In your class, it is customary to joke about someone so that the whole class laughs	0.694 (0.014)	<0.001
	In your class, it is customary to fight	0.803 (0.014)	<0.001
	In your class, it is customary to call names	0.92 (0.009)	<0.001
	In your class, it is customary to interfere with each other, make nervous, molest	0.821 (0.012)	<0.001
	When there is a fight at school, you don't pay attention, it's a common thing	0.618 (0.017)	<0.001

Correlations between factors were −0.634 between School Well-Being and Deviant Behavior; −0.669 between School Well-Being and Subjective Unsafety; and 0.791 between Deviant Behavior and Subjective Unsafety.

### Convergent Validity

Correlations between SCQ and the measures of victimization, aggression, mental wellbeing, and types of academic motivation are presented in Table 3.

**Table 3:** Correlations (Pearson's *r*) between three scales of SCQ and the measures of victimization, aggression, mental wellbeing, and types of academic motivation.

**Scales**	***n* ^A^**	**Deviant behavior**	**School wellbeing**	**Subjective unsafety**
Victimization	3,380	0.220***	−0.212***	0.289***
Aggression	3,279	0.171***	−0.153***	0.234***
Mental Well-being	195	−0.184*	0.509***	−0.187**
Intrinsic Motivation to Know	195	−0.288***	0.439***	−0.311***
Intrinsic Motivation to Accomplish Things	195	−0.157*	0.359***	−0.207**
Intrinsic Motivation to Experience Stimulation	195	−0.223**	0.415***	−0.236***
Identified Regulation	195	−0.073	0.334***	−0.125
Introjected Motivation	195	0.038	0.030	−0.040
External Motivation	195	0.201**	−0.208**	0.169*
Amotivation	195	0.295***	−0.324***	0.294***

***p <0.001; **p <0.01; *p <0.05.

*^a^n is the number of respondents who fill both questionnaires*.

Due to the large sample size, we did not discuss all the significant correlations, but those whose absolute values exceed 0.2 are discussed. The results revealed weak but significant correlations between the Deviant Behavior scale and scales of Victimization, Aggression, External Motivation, and Amotivation (positive); Intrinsic Motivation to Know and Intrinsic Motivation to Experience Stimulation (negative).

The School Well-Being scale demonstrated a moderate positive correlation with the scales of Mental Well-Being, Intrinsic Motivation to Know, Intrinsic Motivation to Accomplish Things, Intrinsic Motivation to Experience Stimulation, and Identified Regulation; moderate negative correlations with the scales of Amotivation; and weak negative correlation with the scales of Victimization, Aggression, and External Motivation.

Subjective unsafety correlates weakly but significantly with the scales of Victimization, Aggression, External Motivation, and Amotivation (positive) and with the scales of Intrinsic Motivation to Know, Intrinsic Motivation to Accomplish Things, and Intrinsic Motivation to Experience Stimulation (negative).

In general, these results demonstrate an acceptable convergent validity of the new scales and the measures of victimization, aggression, mental wellbeing, and academic motivation.

## Discussion

The current paper is dedicated to the development and validation of a new tool for assessing the school climate, which is titled SCQ and includes 22 items and three scales. There was no reliable, valid, convenient, and short instrument to assess the quality of the school climate by the school students in Russia, so this tool was designed to fill this shortfall. The availability of such a tool is necessary for the different research studies on the educational environment (school students' wellbeing, their academic engagement or burnout, classroom relationships, classroom management, etc.); it may be useful for designing prevention programs and the performance of psychological counseling within the school.

The correlational research included checking the reliability and structural and convergent validity. Both hypotheses about the psychometric properties of SCQ were supported. As expected, SCQ has a three-factorial structure, according to CFA. The reliability of the tool is satisfactory (α is from 0.59 to 0.72). This result suggests that the developed questionnaire allows us to assess three factors of the school climate—Deviant Behavior, School Well-Being, and Subjective Unsafety. Deviant Behavior is a component related to the peculiarities of compliance with social norms, which allows the school to create a more or less comfortable atmosphere in the classroom and at the school. School Well-Being is a factor related to perceived psychological wellbeing. The third factor—Subjective Unsafety—reflects a sense of security (or lack thereof) in the social space of the school. These three factors are expected to correlate with each other, which is a consequence of their interdependence. For example, a high level of Deviant Behavior can also cause a feeling of insecurity at school. Nevertheless, it seems to us important to separate these three components for a more comprehensive assessment of the school climate.

Furthermore, we have revealed clear results regarding convergent validity. All three scales of SCQ correlate with the scales of Victimization and Aggression from the Revised Olweus Bully/Victim Questionnaire, according to Astor et al. (2002), Espelage and Swearer (2003), Kosciw et al. (2011), Hong and Espelage (2012), Thapa et al. (2013), and Berkowitz et al. (2017). The three scales describe the positive, resource side of the school climate (School Well-Being scale) and the aspects of internal (e.g., anxiety, depression, low self-esteem, and victimization) and external (e.g., aggression, bullying) problems (Subjective Unsafety and Deviant Behavior scales, respectively).

Our results reveal significant correlations between the SCQ scales and the scales of different types of Academic Motivation, which is in line with Berkowitz et al. (2017), Volk (2020), and Wang et al. (2020), and others. The perception of the school climate significantly correlates with the different types of the academic motivation, the School Well-Being scale is associated with intrinsic motivation, whereas the Subjective Unsafety and Deviant Behavior scales are associated with external motivation and amotivation.

Finally, the scale of School Well-Being has a strong positive correlation with a scale of Mental Well-Being, whereas the scales of Deviant Behavior and Subjective Unsafety have weak but significant negative correlations with it. This corresponds with the results by Aldridge et al. (2016); Lombardi et al. (2019); and the large number of studies confirming the associations between a positive school climate and a reduction in disruptive behavior and an improvement in subjective wellbeing and life satisfaction. These results give evidence for the good divergent validity of SCQ.

All these results give evidence for the good divergent validity of the SCQ. To sum up, we developed an instrument to measure several aspects of school climate in Russia, and this new tool widely extends the opportunities for research in the field of educational psychology. It can be used both to study the specifics of the school climate in different regions of the same country and for cross-cultural comparisons.

## Conclusion

This paper reports that the correlational validation study results in a valid, reliable, and convenient 22-item instrument to measure the school climate from the school students' perspective. As expected, the SCQ has a three-factorial structure. In line with the results obtained in other research, the scales of SCQ correlate with victimization, aggression, mental wellbeing, and academic motivation indicators. It may be concluded that the aim of our research has been achieved.

The current study is not free of limitations; the most important of them could be overcome by adding some more objective information about the school environments and by adding measures of the school climate from the perspective of the school staff (Olsen et al., 2018). Nevertheless, the new instrument can be recommended for psychological research related to school students' wellbeing, burnout, stress, school adjustment, and other issues in the area of educational psychology.

## Data Availability Statement

The datasets generated during and/or analyzed during the current study are available from the corresponding author on reasonable request.

## Ethics Statement

The studies involving human participants were reviewed and approved by National Research University Higher School of Economics Committee on Interuniversity Surveys and Ethical Assessment of Empirical Research. Written informed consent to participate in this study was provided by the participants' legal guardian/next of kin.

## Author Contributions

AB developed the main idea of the paper, collected the data, organized the database, wrote the first draft of the manuscript, contributed to the manuscript revision, read, and approved the submitted version. AK contributed to the study's conception and design, performed the statistical analysis, contributed to the manuscript revision, read, and approved the submitted version. KK contributed to the study's conception and design, collected the data, read, and approved the submitted version. All authors approved the submitted version of the manuscript.

## Conflict of Interest

The authors declare that the research was conducted in the absence of any commercial or financial relationships that could be construed as a potential conflict of interest.

## Publisher's Note

All claims expressed in this article are solely those of the authors and do not necessarily represent those of their affiliated organizations, or those of the publisher, the editors and the reviewers. Any product that may be evaluated in this article, or claim that may be made by its manufacturer, is not guaranteed or endorsed by the publisher.

## References

[B1] Aldridge J. M. Fraser B. J. Fozdar F. Ala'i K. Earnest J. Afari E. (2016). Students' perceptions of school climate as determinants of wellbeing, resilience and identity. Improv. Sch. 19, 5–26. 10.1177/1365480215612616

[B2] Alliance for the Study of School Climate (2021). School Climate Quality Analytic Assessment Instrument and School-based Evaluation/Leadership Team Assessment Protocol. ASSC. Retrieved from: http://web.calstatela.edu/centers/schoolclimate/assessment/school_survey.html (assessed February 7, 2022).

[B3] Ashiabi G. S. O'Neal K. K. (2015). Child social development in context: an examination of some propositions in Bronfenbrenner's bioecological theory. SAGE Open 5, 1–14. 10.1177/2158244015590840

[B4] Astor R. A. Benbenishty R. Zeira A. Vinokur A. (2002). School climate, observed risky behaviors, and victimization as predictors of high school students' fear and judgments of school violence as a problem. Health Educ. Behav. 29, 716–736. 10.1177/109019802237940 12456131

[B5] Astor R. A. De Pedro K. T. Gilreath T. D. Esqueda M. C. Benbenishty R. (2013). The promotional role of school and community contexts for military students. Clin. Child Fam. Psychol. Rev. 16, 233–244. 10.1007/s10567-013-0139-x 23760904

[B6] Berger C. Brotfeld C. Espelage D. L. (2022). Extracurricular activities and peer relational victimization: role of gender and school social norms. J. Sch. Violence. 20, 611–626. 10.1080/15388220.2022.2026226

[B7] Berkowitz R. Glickman H. Benbenishty R. Ben-Artzi E. Raz T. Lipshtadt N.. (2015). Compensating, mediating, and moderating effects of school climate on academic achievement gaps in Israel. Teach. Coll. Rec. 117, 1–34. 10.1177/016146811511700703

[B8] Berkowitz R. Moore H. Astor R. A. Benbenishty R. (2017). A research synthesis of the associations between socioeconomic background, inequality, school climate, and academic achievement. Rev. Educ. Res. 87, 425–469. 10.3102/0034654316669821

[B9] Birkett M. Espelage D. L. Koenig B. (2009). LGB and questioning students in schools: the moderating effects of homophobic bullying and school climate on negative outcomes. J. Youth Adolesc. 38, 989–1000. 10.1007/s10964-008-9389-1 19636741 PMC6322390

[B10] Bochaver A. A. Kuznetsova V. B. Bianki E. M. Dmitrievskiy P. V. Zavalishina M. A. Kaporskaya N. A.. (2015). The risk of bullying questionnaire. Psychol. Issues 5, 146–157. (In Russ.).

[B11] Bradshaw C. P. Cohen J. Espelage D. L. Nation M. (2021). Addressing school safety through comprehensive school climate approaches. School Psych. Rev. 50, 221–236. 10.1080/2372966X.2021.192632131938876

[B12] Breivik K. Olweus D. (2015). An item response theory analysis of the Olweus Bullying scale. Aggress. Behav. 41, 1–13. 10.1002/ab.21571 27539870

[B13] Bronfenbrenner U (1979). The Ecology of Human Development: Experiments by Nature and Design. Cambridge, MA: Harvard University Press.

[B14] Bronfenbrenner U. Morris P. A. (2006). “The bioecological model of human development,” in Handbook of Child Psychology: Theoretical Models of Human Development, eds R. M. Lerner, and W. Damon (New York; Washington, DC: John Wiley & Sons Inc.), 793–828.

[B15] Brown T. A (2006). Confirmatory Factor Analysis for Applied Research. New York, NY: The Guilford Press, 493.

[B16] Bushina E. V. Muminova A. M. (2021). Adaptation of the revised olweus bully/victim questionnaire — Russian version. Soc. Psychol. Soc. 12, 197–216. 10.17759/sps.2021120212

[B17] Capp G. Astor R. A. Gilreath T. D. (2020). Advancing a conceptual and empirical model of school climate for school staff in California. J. Sch. Violence 19, 107–121. 10.1080/15388220.2018.1532298

[B18] Cohen J. McCabe E. M. Michelli N. M. Pickeral T. (2009). School climate: research, policy, practice, and teacher education. Teach. Coll. Rec. 111, 180–213. 10.1177/016146810911100108

[B19] Cronbach L. J (1951). Coefficient alpha and the internal structure of tests. Psychometrika 16, 297–334. 10.1007/BF02310555

[B20] Crosnoe R (2011). “Schools, peers, and the big picture of adolescent development,” in Adolescent Vulnerabilities and Opportunities: Developmental and Constructivist Perspectives, eds E. Amsel, and J. Smetana (New York, NY: Cambridge University Press), 182–204.

[B21] Demirtas-Zorbaz S. Akin-Arikan C. Terzi R. (2021). Does school climate that includes students' views deliver academic achievement? A multilevel meta-analysis. Sch. Effect. Sch. Improv. 32, 543–563. 10.1080/09243453.2021.1920432

[B22] Ebbert A. M. Luthar S. S. (2021). Influential domains of school climate fostering resilience in high achieving schools. Int. J. Sch. Educ. Psychol. 9, 305–317. 10.1080/21683603.2021.1898501

[B23] Espelage D. L. Swearer S. M. (2003). Research on school bullying and victimization: what have we learned and where do we go from here? Sch. Psych. Rev. 32, 365–383. 10.1080/02796015.2003.12086206

[B24] Farina K. A (2019). Promoting a culture of bullying: understanding the role of school climate and school sector. J. Sch. Choice 13, 94–120. 10.1080/15582159.2018.1526615

[B25] Freudenberg N. Ruglis J. (2007). Reframing school dropout as a public health issue. Prev. Chronic Dis. 4, A107. 17875251 PMC2099272

[B26] Gage N. A. Larson A. Chafouleas S. M. (2016). The meriden school climate survey-student version: preliminary evidence of reliability and validity. Assess. Effect. Interven. 41, 67–78. 10.1177/1534508415596960

[B27] Gordeeva T. O. Sychev O. A. Osin E. N. (2014). Oprosnik ≪Shkaly akademicheskoy motivacii≫ = The ≪Academic motivation scales≫ questionnaire. Psychol. J. 35, 96–107. (In Russ.)

[B28] Gottfredson G. D. Gottfredson D. C. Payne A. A. Gottfredson N. C. (2005). School climate predictors of school disorder: results from a national study of delinquency prevention in schools. J. Res. Crime Delinq. 42, 412–444. 10.1177/0022427804271931

[B29] Grazia V. Molinari M. (2021). School climate multidimensionality and measurement: a systematic literature review. Res. Papers Educ. 36, 561–587. 10.1080/02671522.2019.1697735

[B30] Hong J. S. Espelage D. L. (2012). A review of research on bullying and peer victimization in school: an ecological system analysis. Aggress. Violent Behav. 17, 311–322. 10.1016/j.avb.2012.03.003

[B31] Khlomov K. D. Bochaver A. A. Korneev A. A. (2021). Well-being and coping with stress among Russian adolescents in different educational environments. Psychol. Russia State Art 14, 68–80. 10.11621/pir.2021.0305 PMC9888054 36733536

[B32] Kosciw J. G. Greytak E. A. Bartkiewicz M. J. Boesen M. J. Palmer N. A. (2011). The 2011 National School Climate Survey: The Experiences of Lesbian, Gay, Bisexual and Transgender Youth in Our Nation's Schools. Gay, Lesbian and Straight Education Network (GLSEN). Retrieved from: http://eric.ed.gov/?id=ED535177 (assessed February 7, 2022).

[B33] La Salle T. P. Meyers J. Varjas K. Roach A. (2015). A cultural-ecological model of school climate. Int. Jo. Sch. Educ. Psychol. 3, 157–166. 10.1080/21683603.2015.104755029154406

[B34] Lombardi E. Traficante D. Bettoni R. Offredi I. Giorgetti M. Vernice M. (2019). The impact of school climate on well-being experience and school engagement: a study with high-school students. Front. Psychol. 10, 2482. 10.3389/fpsyg.2019.02482 31749747 PMC6848455

[B35] National School Climate Center (2021). Measuring School Climate (CSCI). Retrieved from: https://schoolclimate.org/services/measuring-school-climate-csci/csci-report/ (assessed February 7, 2022).

[B36] O'Brennan L. Bradshaw C. P. (2017). “The transactional association between school climate and bullying,” in Handbook of Bullying Prevention: A Life Course Perspective, ed C. Bradshaw (New York; Washington, DC: National Association of Social Workers Press), 165–176.

[B37] Olsen J. Preston A. I. Algozzine B. Algozzine K. Cusumano D. (2018). A review and analysis of selected school climate measures. Clear. House 91, 47–58. 10.1080/00098655.2017.1385999

[B38] R Core Team (2020). R: A Language and Environment for Statistical Computing. Vienna: R Foundation for Statistical Computing.

[B39] Reaves S. McMahon S. D. Duffy S. N. Ruiz L. (2018). The test of time: a meta-analytic review of the relation between school climate and problem behavior. Aggress. Violent Behav. 39, 100–108. 10.1016/j.avb.2018.01.006

[B40] Robinson O. C, Lopez, F. G. Ramos K. Nartova-Bochaver S. (2013). Authenticity, social context, and wellbeing in the United States, England, and Russia: a three country comparative analysis. J. Cross Cult. Psychol. 44, 719–737. 10.1177/0022022112465672

[B41] Rosseel Y (2012). lavaan: an R Package for structural equation modeling. J. Stat. Softw. 48, 1–36. 10.18637/jss.v048.i0225601849

[B42] Tennant R. Hiller L. Fishwick R. Platt S. Joseph S. Weich S.. (2007). The Warwick-Edinburgh mental well-being scale (WEMWBS): development and UK validation. Health Qual. Life Outcomes 5, 63. 10.1186/1477-7525-5-63 18042300 PMC2222612

[B43] Thapa A. Cohen J. Guffey S. Higgins-D'Alessandro A. (2013). A review of school climate research. Rev. Educ. Res. 83, 357–385. 10.3102/0034654313483907

[B44] U.S. Department of Education Office of Safe Healthy Students (2016). Quick Guide on Making School Climate Improvements. Retrieved from: http://safesupportivelearning.ed.gov/SCIRP/Quick-Guide (assessed February 7, 2022).

[B45] Vallerand R. J. Pelletier L. G. Blais M. R. Brière N. M. Sénécal C. Vallières E. F. (1992). The academic motivation scale: a measure of intrinsic, extrinsic and amotivation in education. Educ. Psychol. Meas. 52, 1003–1017. 10.1177/0013164492052004025

[B46] Volk T (2020). An Examination of the Relationship Between School Climate, Self-Determined Academic Motivation, and Academic Outcomes Among Middle and High School Students [Dissertation]. USA: University of Connecticut, 2435.

[B47] Wang M.-T. Degol J. L. (2016). School climate: a review of the construct, measurement, and impact on student outcomes. Educ. Psychol. Rev. 28, 315–352. 10.1007/s10648-015-9319-1

[B48] Wang Q. Lee K. C. S. Hoque K. E. (2020). The effect of classroom climate on academic motivation mediated by academic self-efficacy in a higher education institute in China. Int. J. Lear. Teach. Educ. Res. 19, 8. 10.26803/ijlter.19.8.11

[B49] WestEd (2022). California School Climate, Health, and Learning Surveys. Retrieved from: https://calschls.org/ (assessed February 7, 2022).

[B50] Yoon J. Sulkowski M.L. Bauman S.A. (2016). Teachers' responses to bullying incidents: effects of teacher characteristics and contexts. J. Sch. Violence 15, 91–113. 10.1080/15388220.2014.963592

[B51] Zullig K. J. Koopman T. M. Patton J. M. Ubbes V. A. (2010). School climate: historical review, instrument development, and school assessment. J. Psychoeduc. Assess. 28, 139–152. 10.1177/0734282909344205

[B52] Zysberg L. Schwabsky N. (2021). School climate, academic self-efficacy and student achievement. Educ. Psychol. 41, 467–482. 10.1080/01443410.2020.1813690

