# Supplementary material for: School Climate Questionnaire: A New Tool for Assessing the School Environment
Source: Front Psychol. 2022 Jul 1;13:871466. doi: 10.3389/fpsyg.2022.871466 (PMC9286750; doi:10.3389/fpsyg.2022.871466)
Supplement: Supplementary file 1 [file Data_Sheet_1.docx]

**Appendix 1.**

The *School Climate Questionnaire*: Corresponding English and Russian versions

**Инструкция:** Мы предлагаем вам поучаствовать в исследовании, потому что нам хочется понять, насколько комфортно ученики себя ощущают в школе. Исследование анонимно, никто не сможет узнать, чьи это ответы. Здесь нет правильных или неправильных ответов. Мы понимаем, что люди в школах не только учат и учатся, но и общаются, дружат, дерутся, спорят, делают что-то вместе. Нам как раз интересно, насколько приятно и легко в школе с кем-то взаимодействовать, или тяжело и сложно. Спасибо, что принимаете участие в нашем исследовании. Отметьте, пожалуйста, для каждого пункта, согласны ли вы или не согласны с этими утверждениями.

**Instruction:** We invite you to participate in this study, because we want to understand how comfortable students feel at school. The research is anonymous, no one will be able to find out whose answers these are. There are no right or wrong answers here. We understand that people in schools not only teach and study, but also communicate, make friends, fight, argue, do something together. We are just wondering how pleasant and easy it is to interact with someone at school, or how hard and difficult it is. Thank you for taking part in our research. Please mark for each item whether you agree or disagree with these statements.

| Item code | English wording | Russian wording |
| --- | --- | --- |
| 1 | There's someone in your class that even a teacher can't handle | В вашем классе есть кто-то, с кем даже учитель не может справиться |
| 2 | At your school, swearing sounds during recess in personal conversations | В вашей школе мат, ругательства звучат на переменах в личных разговорах |
| 3 | In your school, swearing is not accepted at all | В вашей школе мат, ругательства не приняты вообще |
| 4 | In your school, they smoke in the lavatories, under the stairs | В вашей школе курят в туалетах, под лестницами |
| 5 | In your school, the walls, furniture are covered with writing, stained | В вашей школе стены, мебель исписанные, испачканные |
| 6 | If someone starts yelling, fighting, the class «go nuts», what does it take to make it stop? The director should come | Если кто-то начинает орать, драться, класс «встает на уши». Что нужно, чтобы это прекратилось? должен прийти директор |
| 7 | If someone starts yelling, fighting, the class «go nuts», what does it take to make it stop? It will stop when everyone gets tired | Если кто-то начинает орать, драться, класс «встает на уши». Что нужно, чтобы это прекратилось? это прекратится, когда все устанут |
| 8 | Your class has a reputation of bullies | Ваш класс имеет репутацию хулиганов |
| 9 | I try not to carry valuables to school at all | Ценные вещи стараюсь не носить в школу вообще |
| 10 | In your class, it is customary to have fun together after the lessons | В вашем классе принято вместе развлекаться после уроков |
| 11 | In your class, it is customary to stand up for your own | В вашем классе принято заступаться за своих |
| 12 | In your class, it is customary not to interfere with each other to do what you want | В вашем классе принято не мешать друг другу заниматься, чем захочется |
| 13 | There is someone in your class that everyone respects | В вашем классе есть кто-то кого все уважают |
| 14 | If someone starts yelling, fighting, the class «go nuts». What does it take to make it stop? Оne of the students should say «That’s enough» | Если кто-то начинает орать, драться, класс «встает на уши». Что нужно, чтобы это прекратилось? кто-то из учеников должен сказать «хватит» |
| 15 | You generally like your school, it's comfortable, there is interesting | В школе вам в целом нравится, приятно, интересно |
| 16 | You don't like school in general, it's uncomfortable, no one is friends with anyone | В школе вам в целом не нравится, плохо, никто ни с кем не дружит |
| 17 | Your class has a reputation of the honor students | Ваш класс имеет репутацию отличников |
| 18 | In your class, it is customary to joke about someone so that the whole class laughs | В вашем классе принято шутить над кем-нибудь так, чтобы смеялся весь класс |
| 19 | In your class, it is customary to fight | В вашем классе принято драться |
| 20 | In your class, it is customary to call names | В вашем классе принято обзываться |
| 21 | In your class, it is customary to interfere with each other, make nervous, molest | В вашем классе принято мешать друг другу, лезть, приставать |
| 22 | When there is a fight at school, you don't pay attention, it's a common thing | Когда в школе происходит драка, вы не обращаете внимания, это обычное дело |
